# Coregistration of eye movements and EEG reveals frequency effects of words and their constituent characters in natural silent Chinese reading

**DOI:** 10.1038/s41598-024-82817-6

**Published:** 2025-01-13

**Authors:** Taishen Zeng, Longxia Lou, Zhi-Fang Liu, Chaoyang Chen, Zhijun Zhang

**Affiliations:** 1 Department of Psychology, Hangzhou Normal University, 2318 Yuhangtang Road, Hangzhou City, 311121 Zhejiang Province China; 2 Department of Psychology, Ningbo University, Ningbo, 315211 China; 3 Department of Psychology and Behavioral Sciences, Zhejiang University, Hangzhou, 310058 China

**Keywords:** Chinese reading, Word frequency effects, Character frequency effects, Fixation-related potential, Language, Reading, Human behaviour

## Abstract

We conducted two experiments to examine the lexical and sub-lexical processing of Chinese two-character words in reading. We used a co-registration electroencephalogram (EEG) for the first fixation on target words. In Experiment 1, whole-word occurrence frequency and initial constituent character frequency were orthogonally manipulated, while in Experiment 2, whole-word occurrence frequency and end constituent character frequency were orthogonally manipulated. Results showed that word frequency facilitated eye-tracking measures, while initial and end character frequencies inhibited them. Classical word frequency effects on N170 and N400 in the posterior region and reversed word frequency effects over the anterior region were consistently observed in both experiments. Experiment 1 revealed an inhibiting effect of initial character frequency on anterior N170. In Experiment 2, interaction between end-character frequency and word frequency showed reliable effects on anterior N170 and N400. These results demonstrate both facilitating and inhibiting word frequency effects, along with inhibiting effects of character frequency and that word frequency moderates the inhibiting effects of end constituent character frequency during natural silent Chinese reading.

## Introduction

Efficient, automatic word decoding is crucial for text comprehension. This study investigates lexical and sub-lexical processing in Chinese reading. A common approach for dissociating these processes involves manipulating lexical and sub-lexical frequency factors^1,2^. Numerous studies have consistently revealed facilitating word frequency effects in both lexical decision tasks and reading^3–5^. Findings show that readers of alphabetic languages fixate on higher-frequency words for a shorter time and skip them more often than the lower-frequency ones during reading^6,7^. Event-related potential (ERP) studies also revealed that word frequency decreases the amplitude of the N170 and N400 components^8–14^, which have been interpreted as promoting word form access and facilitating semantic access, respectively.

Research indicates that sub-lexical factors impact word processing. Specifically, studies consistently reveal inhibitory syllable frequency effects, with increased recognition time for high first syllable frequency words compared to low first syllable frequency words in orthographic transparent scripts, such as German, French, and Spanish^15–19^. ERP studies have shown that first syllable frequency increases N400 amplitude over the anterior scalp regions^8,20^. These inhibiting effects are more pronounced for low lexical frequency words, interpreted as greater activation of syllabic neighbors in high first frequency syllable words compared to low-frequency syllables^3,21^. Importantly, these inhibitory effects of syllable frequency are language-dependent. Specifically, in opaque orthographic scripts, i.e., English, facilitating first and end syllable frequency effects were reported in word naming and lexical decision tasks^22^. Croot et al. reported facilitating mean syllable frequency effects on production tasks^23^. These effects were interpreted as resulting from poor syllable boundary cues that preclude the activation of syllabic neighbors before the target is recognized.

As a non-alphabetical script, the basic writing unit of Chinese is the character, which are square-shaped and directly linked to a monosyllabic sound. Most Chinese words consist of multiple characters, with approximately 72% comprising two characters^24^, and there are no spaces marking word boundaries in Chinese written scripts. The facilitating effects of whole word frequency among two-character words have been replicated multiple times. It has been observed that Chinese readers, including children, young, and older adults, fixate on frequent words for shorter durations and skip them more often than infrequent words^24–27^. Using a rapid serial visual presentation (RSVP) paradigm, in which words are displayed one at a time, Lee et al. examined word frequency effects in traditional Chinese reading and found that word frequency reduces the amplitudes of N100 and N400 ^28^.

It is theoretically valuable to examine the effects of character frequency, as Chinese is an opaque orthographic script characterized by clear boundaries but low spelling sound correspondence. Surprisingly, the role of constituent character frequency in two-character word decoding remains a topic of ongoing debate. In their regression analysis, Li et al. did not find evident effects of constituent character frequency in simplified Chinese reading^24^. Conversely, Ma et al. observed that the initial constituent character frequency of words decreased the fixation duration on pre-target words, thereby supporting the facilitating effect viewpoint^26^. In contrast, Yu et al. reported inhibitory effects of initial constituent character frequency effects on fixation duration^29^. The inhibitory effects of initial character frequency in lexical decision and reading tasks were replicated by Xiong et al., although facilitating effects were observed in word naming tasks^30^.

ERP data regarding the impacts of constituent character frequency on Chinese multi-character word processing are surprisingly sparse. Huang et al. revealed that words with large neighborhoods and high-frequency neighbors elicited greater effects on N400 than those without high-frequency neighbors; this difference was not observed for words with small neighborhoods^31^. They speculated that all the words sharing the same constituent character as the target word begin to activate during early word recognition stages. In contrast, low-frequency words in their neighborhoods face less competition in later word recognition stages, thus producing inhibitory effects. Subsequently, these inhibiting effects were replicated in two MEgastudy of Lexical Decision datasets, which reported that both high frequency of initial and end characters increase recognition time for two-character words^32^, and that character contextual diversity enhances the N400 ^33^.

Experimental methods were employed to disentangle the effects of constituent character frequency from those of whole word frequency effects in reading, as well as to examine how word frequency moderates character effects. In an early eye-tracking study, Yan et al. orthogonally manipulated whole word frequency and its initial and end constituent character frequencies^25^. The results revealed facilitating effects of both initial and end constituent character frequency, indicating that high constituent character frequency reduced the viewing time for the target word. Furthermore, the constituent character frequency effects were diminished by whole word frequency. In another study, in which the initial and end constituent characters frequency of two-character words were manipulated orthogonally, no reliable character frequency effects were observed for high frequent words; however, for infrequent words, the initial characters frequency decreased fixation time measures on the end character^34^. Conversely, no evidence supported the viewpoint that word frequency moderates the effects of initial constituent character frequency in two subsequent studies^29,30^.

With high time resolutions, electroencephalogram (EEG) data are well-suited for examining lexical and sub-lexical processing. However, how conclusions drawn from ERP studies on lexical decision tasks can be generalized to natural reading remains unclear. Substantial divergences have been revealed between lexical decision and reading^31,35^. In reading, word frequency effects vanished when word presentation rates were similar to natural salient reading speeds^36^. Additionally, RSVP paradigms used in EPR studies may limit their ecological validity. Despite their good ecological validity, dissociating lexical and sub-lexical processing based solely on eye tracking measures proves challenging. Therefore, combining eye tracking with ERP recording is a better method for exploring lexical and sub-lexical processing in natural silent reading compared to using either method alone^39^. Co-registration of EEG data with eye tracking has recently become well-established and developed^37,38^, providing an alternative means to investigate the natural reading process. However, co-registration studies focusing on Chinese multi-character words and their constituent character processing during natural silent reading remain scarce.

In the present study, the roles of constituent character frequency, namely facilitating or inhibiting effects, on word processing in natural salient Chinese reading were examined through co-registration of EEG data with eye movements. Given that the impacts from initial and end characters on word processing are slightly different^40^, two experiments were conducted. In Experiment 1, whole words and their initial character frequencies were orthogonally manipulated, while in Experiment 2, whole words and their end character frequencies were orthogonally manipulated. The facilitating account predicts decreased effects of constituent character frequency on both EEG and eye tracking data, while the inhibiting account predicts increased impacts from character frequency. Additionally, we consider how word frequency moderates the effects of constituent character frequency.

## Methods

### Ethical consideration

The project, titled “Study on the cognitive mechanisms of special population in Chinese reading,” received ethical approval from the Cognition and Brain Disorders Research Centre at our university, where the experiment was conducted. The Institutional Review Board (IRB) protocol number of this approval is 20190408. All methods were conducted in accordance with relevant guidelines, regulations, and data were collected anonymously. The participants provided written informed consent by signing a form before the experiments. All methods were carried out in accordance with the Declaration of Helsinki.

### Participants

We used the G*Power 3.1 to calculate the required sample size, referencing a previous study that orthogonally manipulated whole-word and constituent character frequency factors^25^. We aimed to investigate how word frequency moderates the effects of its constituent character frequency. The interaction effect size of word frequency with its constituent character frequency was derived from variance analysis results, estimating an effect size on gaze duration of *d* = 0.20. Therefore, we assumed a small effect size (*d* = 0.20) and aimed for a minimum sample size of 55 for a within-subjects design with 95% power (1-*β*) and (*α* = 0.05). Consequently, the sample size for our experiments included 56 college students (36 female and 20 male). The same participants performed both Experiments 1 and 2, with the order counterbalanced across participants. Half of the participants completed Experiment 1 first, followed by Experiment 2, while the other half completed Experiment 2 first, followed by Experiment 1. Participants took a 30-minute break between the two experiments to prevent fatigue from impacting experimental results. All participants were native Chinese speakers studying at our university and aged 19–25 years (*M* = 19.70, standard deviation *SD* = 0.99). They were right-handed and compensated ¥150 for their participation. Before the experiment, corrected vision was measured using the Tumbling E acuity chart to ensure all the participants had normal vision (M = 4.97, Standard deviation *SD* = 0.09).

### Apparatus and procedure

We recorded EEG signals using a ductile cap with mounted 30 Ag/AgCl electrodes and used a BrainAmp amplifier to magnify the EEG signals to 1000 Hz. The AFz electrode was used as the ground reference. Horizontal and vertical electrooculograms (EOGs) were recorded to correct for eye blinks. All 30 electrodes were kept under 5 kΩ. EOG and EEG data were recorded with a band-pass from AC 0.1–100 Hz. Eye movements were captured with EyeLink 1000 Plus Desktop device at a 1000 Hz rate. Since there was no head restraint device to fix the head within the desktop device, there was no affection for the frontal electrodes. To minimize head movement and enhance eye tracking quality, a chin rest was used, and participants were instructed to keep their heads as still as possible. Transistor-transistor-logic (TTL) pulses were used to synchronize the eye tracking and EEG recording systems online. These TTL pulses were generated by the stimuli display computer. At the beginning and end of each trial, TTL pulses were sent from the stimuli display computer to the BrainAmp amplifier.

Prior to the experiment, all of the participants underwent a visual acuity test. They were seated 60 cm away from the stimuli display computer monitor, a 19-inch LCD device with a resolution of 1024 × 768-pixel, and a refresh rate of 60 Hz. At the start of the experiment, each participant read the instructions and pressed a key to dismiss the instruction screen, followed by a three-point horizontal calibration and validation procedures. During these procedures, participants were instructed to fixate on dots along the horizontal line presented with the sentences. The sentences were displayed one at a time, and the participants were asked to read them for comprehension. Before each trial, the drift calibration accuracy was automatically checked, after which the sentences were displayed. The stimuli were rendered in 20-point Song font on a white background, with each character subtended by approximately 1°. Participants were instructed to press a button once they had finished reading the sentence. Questions appeared randomly after 25% of the sentences, and participants were instructed to respond by pressing the right and left arrow buttons on the keyboard. The experimenter monitored the drift calibration accuracy and re-calibrated the eye tracking device if it exceeded 0.5°. Ten practice trials were conducted at the beginning of the experimental session to familiarize participants with the experimental procedure. The entire session lasted 2 h for each participant, with a break provided after each experiment.

### Data analysis

The mean accuracy for comprehension questions during Experiments 1 and 2 was 94.96% and 96.88%, respectively, with all participants achieving comprehension accuracy above 80%, suggesting that they engaged with the reading task conscientiously. First fixation duration, gaze duration, skipping probability, and re-fixation probability on the target word were analyzed using a linear mixed effects model in R (vision R-4.2.1). First fixation durations and gaze durations shorter than 80 ms or longer than 1200 ms (less than 2% of all fixations) were removed. A maximal random effects structure was employed, treating participants and stimuli as crossed random effects^41^. Continuous dependent variables were analyzed using the LMM package (version 1.1–31), while binary dependent variables were analyzed using the GLMM package (version 3.1-3). Word frequency, character frequency, their interactions, and covariant variables (initial and end character orthographic neighbor sizes) were included as fixed effect factors. We removed the covariant variables when analyzing the probability measures of skipping and re-fixation due to the absence of convergence. Models were fitted using the lme4 package, and *p*-values were estimated with the lmerTest package (version 3.1-3). Log-transformed continuous data were analyzed, yielding similar results to the non-transformed data^42^; therefore, only statistical result values from non-transformed data were reported, including regression coefficients (*b*), standard errors (*SE*), *t* (*t* = *b/SE*) or *Z *(*Z* = *b/SE*) and *p* values.

Fixation-related triggers for the EEG were time-locked to the onset of the initial fixation. Trials in which the reader skipped the target words during first-pass reading were automatically excluded from data analysis. Offline raw EEG data were filtered using a band-pass filter at 0.1–40 Hz. The trials were corrected for eye-movement artifacts using the independent component analysis (ICA) method developed specifically for free viewing studies^43^. We adhered closely to the criterion set by Dimigen. Specifically, spike potentials were overweighted by copying the data from − 20 to 10 ms around the saccades. ICA components whose variance during saccades was over 10% higher than the variance during fixations were removed. Before averaging, an automatic artifact rejection procedure was conducted to remove excessive artifacts from ocular behaviors (exceeding ± 100 µV). The mean fixation-related potentials (FRPs) were calculated from 200 ms prior to the initial fixation to 1000 ms after fixation onset. We epoched and baseline-corrected offline trial data with a 200 ms per stimulus period. Low-quality data were excluded from the statistical analysis, and data from four participants were discarded from the analysis in Experiments 1 and 2 because their valid data points in any condition were fewer than seven trials. Mean amplitudes of N170 and N400 were chosen for analysis based on previous studies^10^. The N170 window was defined as the interval between 140 and 200 ms. Kretzschmar et al. identified an N400 peak at 300 ms after fixation onset, which was earlier than that in the RSVP reading^44^. Therefore, in our study, the time interval of the N400 was 200–400 ms.

**Fig. 1:**
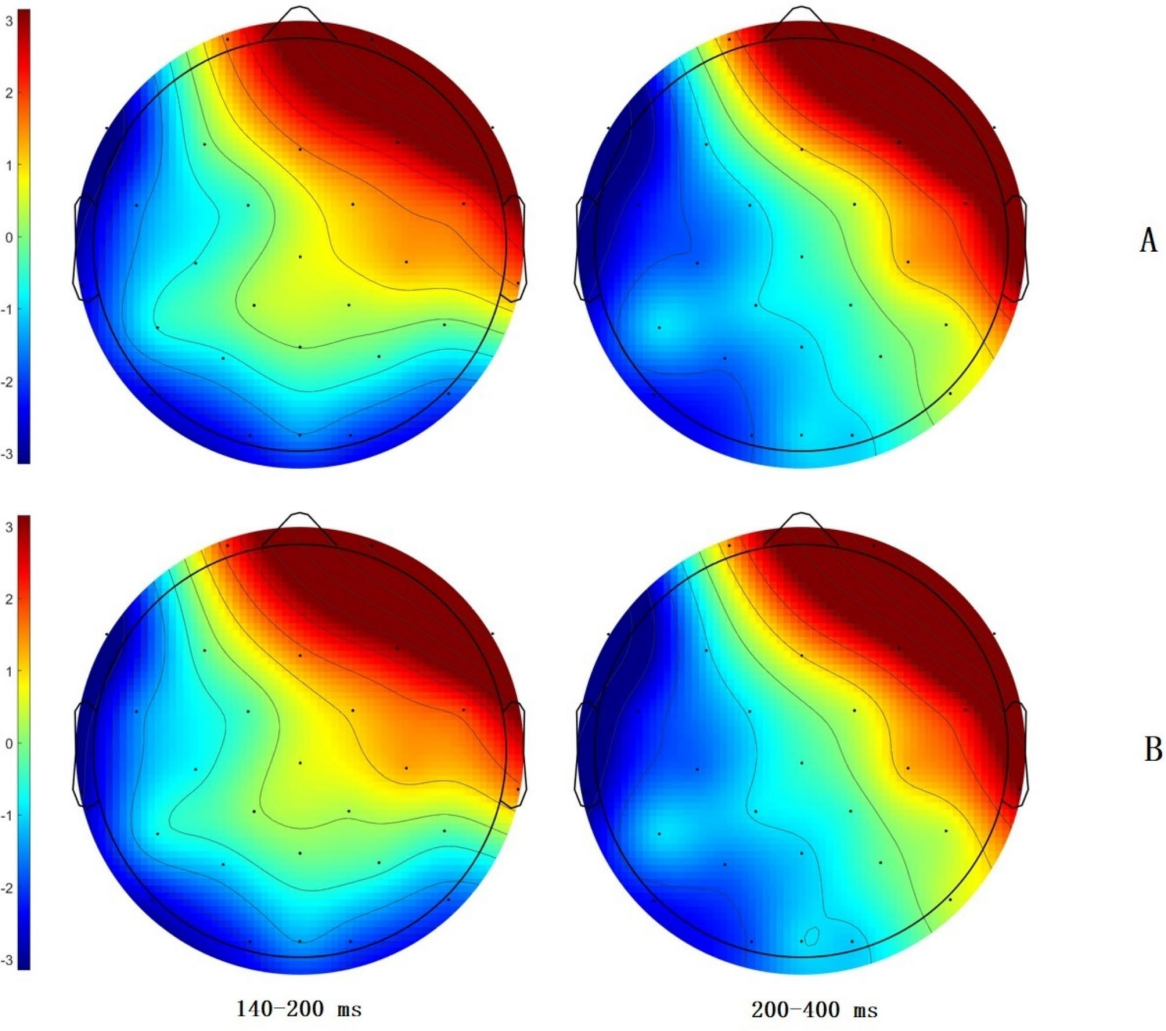
Scalp topographies of the mean FRP amplitudes across four conditions in two time windows for Experiment 1 (**A**) and Experiment 2 (**B**).

We averaged the waveform across conditions and used the region distribution from the collapsed waveform to define the electrodes used for the non-collapsed data^45^. A noticeable gap between the frontal-central and parietal-occipital scalp regions was observed consistently for both Experiments 1 and 2 (Fig. 1). Consequently, we analyzed the ERP responses over these two scalps regions separately. We chose the electrodes with negativity below the zero µV contour line for N170 and N400. Following this criterion, electrodes F3, F7, FC1, FC5, C3, and T7 in the left frontal-central region and P3, P4, P7, P8, Pz, O1, O2, and Oz in the parietal-occipital region were selected for analyzing N170 effects. F3, F7, FC1, FC5, C3, and T7 were used to check the N400 effects in the frontal-central region, while P3, P4, P7, Pz, Q1, O2, and Oz were used to check these effects in the parietal-occipital regions. We defined two regions of interest (ROIs), and the amplitudes of the electrodes within these ROIs were averaged. A linear mixed-effects model was utilized to analyze the mean amplitude of brain response components, including the random structure, word frequency, character frequency, and their interactions as fixed factor effects.

## Experiment 1

### Design and stimuli

The experiment utilized a 2 (word frequency: low versus high) × 2 (initial constituent character frequency: low versus high) within-subject design. Each target word embedded in the sentence frame was composed of two characters. Our material set contained 160 sentence frames, which could embed any of the following target words: low-frequency low-initial-character (LW-LIC), low-frequency high-initial-character (LW-HIC), high-frequency low-initial-character (HW-LIC), or high-frequency high-initial-character (HW-HIC) in the same location (Table 1). The sentence materials were prepared according to the following steps: First, groups of two-character words were selected and classified as LW-LIC, LW-HIC, HW-LIC, or HW-HIC. Second, sentence frames were constructed for these target words, ensuring the frames could embed any LW-LIC, LW-HIC, HW-LIC, or HW-HIC target word in the same location. Third, cloze tasks were used to evaluate how the reader predicted the target from the sentence frames. Twenty college students who were native Chinese speakers and had not participated in any of our FRP experiments rated the predictability of the target words. Each sentence frame, up to but not including the target word, was presented to these college students, who were tasked with completing these sentence frames. Fourth, another group of 40 college students were asked to rate the naturalness of the materials on a point-point scale from 1 (completely unnatural) to 5 (completely natural).

**Table 1:** Example materials used in experiment 1.

Conditions	Translations of example materials
LW-LIC	Li Ming tries to revise the *aerospace* knowledge in order to pass the entrance examination
LW-HIC	Li Ming tries to revise the *drawing* knowledge in order to pass the entrance examination
HW-LIC	Li Ming tries to revise the *automobile* knowledge in order to pass the entrance examination
HW-HIC	Li Ming tries to revise the *biology* knowledge in order to pass the entrance examination

*LW* low word frequency, *HW* high word frequency, *LIC* low initial constituent character frequency, *HIC* high initial constituent character frequency.

Target words are in italics.

Each sentence can accommodate either a LW-LIC, LW-HIC, HW-LIC or HW-HIC target word.

Frequencies were measured as occurrences per 1,000,000 characters acquired from the SUBTLEX-CH corpus^46^. HW range was 20–1522, LW was 0–9.75, HIC was 800–8812, and LIC was 1–539. The strokes of the initial and end characters, as well as the end character frequency of the target words, were balanced across the four experimental conditions (*ps* > 0.05). The pairs of LW-LIC & LW-HIC and HW-LIC & HW-HIC were matched for word frequency (*ps* > 0.05), and the pairs of LW-LIC & HW-LIC and LW-HIC & HW-HIC were matched for initial character frequency (*ps* > 0.05). Regarding contextual predictability, few participants could predict the target words from the preceding text. There were no differences in cloze scores among the four conditions *F* (1, 636) *=* 1.24, *p =* 0.294. Regarding the naturalness of the materials, the score of each material was higher than 4.5, with no reliable effect among the four conditions *F* (1, 636) *=* 1.264, *p =* 0.286. A description of the lexical and contextual properties of the target words is shown in Table 2.

**Table 2:** Specifications of target words and the naturalness of sentences used in experiment 1.

Conditions	Word frequency	First character frequency	Second character frequency	First character strokes	Second character strokes	Cloze	Sentences naturalness
LW-LIC	2 (1)	116 (131)	1012 (1300)	7.64 (2.01)	7.57 (2.44)	0.09 (0.68)	4.82 (0.13)
LW-HIC	2 (2)	2146 (1725)	1025 (1415)	7.36 (2.44)	7.59 (2.72)	0.09 (0.68)	4.82 (0.15)
HW-LIC	88 (75)	204 (120)	1113 (1676)	7.50 (2.07)	7.48 (2.59)	0.25 (1.09)	4.84 (0.13)
HW-HIC	88 (134)	2052 (1445)	1123 (1397)	7.45 (2.74)	7.65 (2.80)	0.22 (1.17)	4.81 (0.10)

Mean values are shown with standard deviations in parentheses. Frequency in occurrences per million characters.

In total, there are 640 combinations (160 frame sentences × 4 kinds of target words) of experimental stimuli. The Latin square method was used to balance the sentence frames and target word combinations; thus, the combinations were divided into four sets of stimuli, with each set containing 160 frame sentences and an equal number of LW-LIC, LW-HIC, HW-LIC, or HW-HIC target words. Participants were randomly assigned to each list and instructed to read for comprehension. Consequently, stimuli combinations were never repeated for each participant during the experiment. The experimental sentence stimuli in each list were presented randomly and were preceded by 10 practice sentences. In the practice session, five sentences were followed by a comprehension question. Additionally, 40 sentences had comprehension questions in the formal experimental session. The participants were asked to answer these questions by pressing the right and left arrow buttons on the keyboard.

### Results

#### Eye tracking results

The means, *SEs*, and statistical results are presented in Tables 3 and 4. We observed reliable impacts of word frequency on eye tracking behaviors, with low frequency targets being fixated on longer, skipped less often, and refixated more frequently than high frequency words, consistent with previous studies^24–27^. Moreover, reliable initial constituent character frequency effects were also observed, with high initial constituent character frequency words being fixated on longer and refixated more often than low initial character frequency words. These inhibiting effects of initial character frequency were inconsistent with a previous study^25,26^, but consistent with other previous studies^29,30,34^. Non-reliable interactions were observed in any eye-tracking measure.

**Table 3:** The mean and standard errors of eye movement dependent in experiment 1.

Measure	Low frequency word		High frequency word	
	LIC	HIC	LIC	HIC
First fixation duration	249 (2)	248 (2)	236 (2)	237 (2)
Gaze duration	296 (3)	307 (3)	268 (3)	275 (3)
Word skipping probability	16.1 (0.8)	17.9 (0.8)	20.0 (0.8)	21.5 (0.8)
Word refixation probability	18.1 (0.8)	20.4 (0.8)	12.5 (0.8)	14.1 (0.8)

The standard errors are given in parentheses; fixation time measures are in milliseconds; probability measures are in %.

**Table 4:** Statistical results of eye movement dependents in experiment 1.

	First fixation duration				Gaze duration			
	*b*	*SE*	*t*	*p*	*b*	*SE*	*t*	*p*
Intercept	245.432	4.537	54.100	< 0.001	296.133	7.27965	40.680	< 0.001
ICF	− 1.179	2.455	− 0.480	0.631	− 15.065	3.86463	− 3.898	< 0.001
WF	12.125	2.002	6.057	< 0.001	30.743	3.12909	9.825	< 0.001
ICF × WF	2.532	4.005	0.632	0.527	− 3.001	6.260	− 0.479	0.632

	Word skipping probability				Word refixation probability			
	*b*	*SE*	*tZ*	*p*	*b*	*SE*	*Z*	*p*
Intercept	− 1.800	0.147	− 12.220	< 0.001	− 1.848	0.105	− 17.641	< 0.001
ICF	− 0.128	0.058	− 2.204	0.028	− 0.149	0.060	− 2.494	0.013
WF	− 0.282	0.058	− 4.844	< 0.001	0.475	0.060	7.948	< 0.001
ICF × WF	− 0.035	0.116	− 0.304	0.761	− 0.011	0.119	− 0.090	0.928

*ICF* initial character frequency, *WF* word frequency, *ICONS* initial character orthographic neighbors size, *ECONS* end character orthographic neighbors size.

To further support the null interactions, Bayes factor analyses were conducted for the time measures of first fixation duration and gaze duration using the BayesFactor package (version 0.9.12–4.7). The Bayes factors for the full models (*BF*_*Full*_), which included the main word frequency effect, initial constituent character frequency effect, interaction between two factors, and covariant variables of initial and end character orthographic neighbor size, were calculated alongside the models (*BF*_Main_) which contained the main effects of word frequency, initial constituent character frequency effect and the covariant variables. The *BF* values (*BF* = *BF*_Full_/*BF*_main_) were evaluated to test the null interaction hypothesis. A *BF* value less than 1, supports the null interaction hypothesis, indicating a lack of an interactive impact of the two factors. For each reading time measure, a default scale prior (*r* = 0.5) and 100,000 Monte Carlo iterations were used. The Bayesian analysis results for both first fixation duration (*BF =* 4.86 × 10^− 7^) and gaze duration supported the null interaction hypothesis (*BF =* 5.22 × 10^–22^).

#### FRP results

**Fig. 2:**
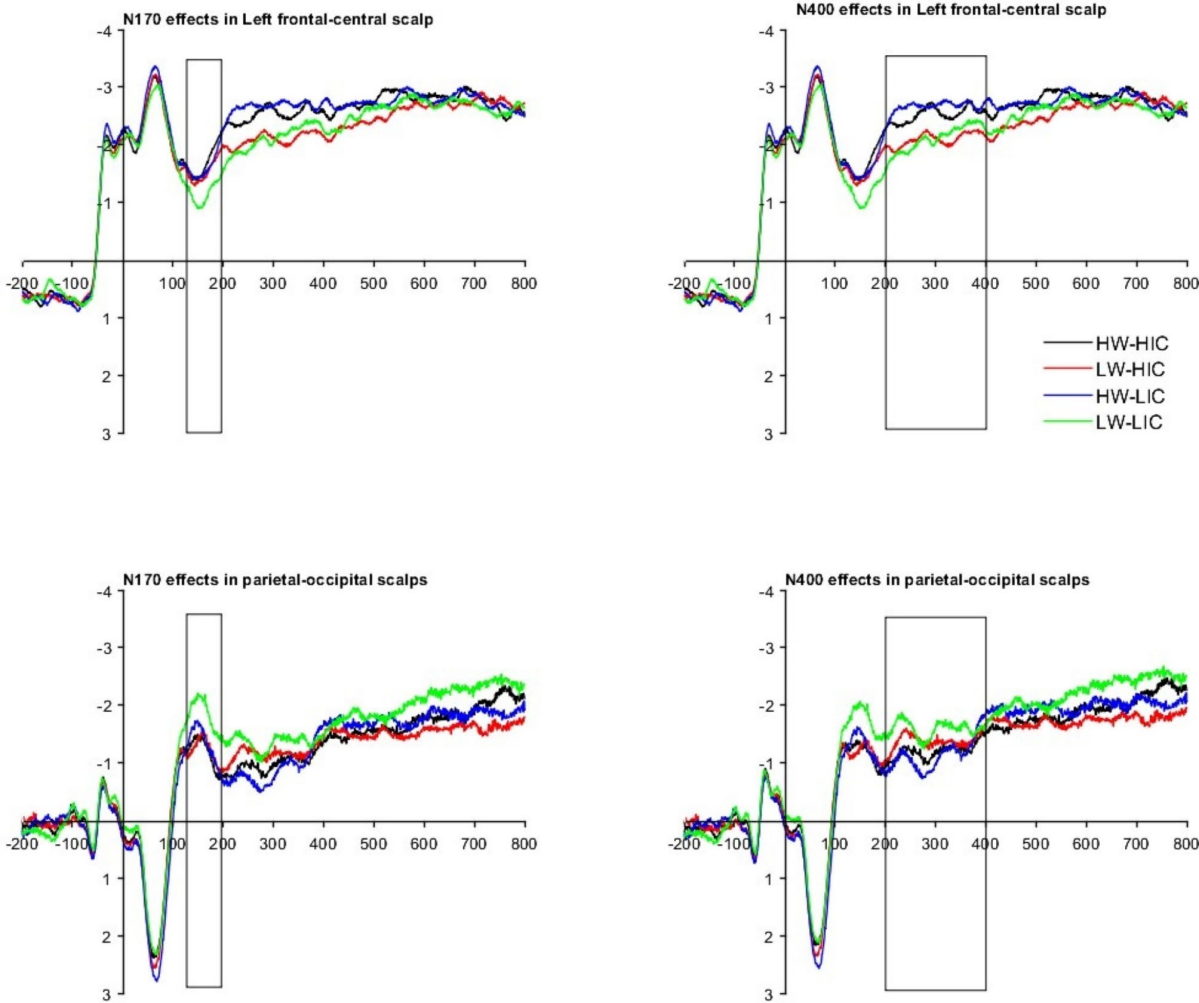
Mean fixation-related potential waveform for four conditions in two interest regions for Experiment 1.

The FRP results are shown in Fig. 2; Tables 5 and 6. Reliable word frequency effects on both N170 and N400 were observed, with low-frequency words eliciting greater N170 and N400 amplitudes in the parietal-occipital region than high-frequency words; however, these word frequency effects were reversed on the electrodes of the left frontal-central region. Reliable or marginal reliable initial constituent character frequency effects were detected on the N170 component, with larger N170 amplitudes in the frontal-central region and smaller N170 amplitudes in the parietal-occipital region, when words with high-frequency initial constituent characters were encountered. The N170 effects of the initial constituent character frequency did not persist in the N400 amplitudes. Non-reliable interactions were observed.

**Table 5:** Mean amplitude standard errors of brain response components in experiment 1.

Measure	Left frontal-central scalp		Parietal-occipital scalp	
	N170	N400	N170	N400
LW-HIC	− 1.544 (0.169)	− 2.085 (0.209)	− 1.092 (0.184)	− 1.267 (0.183)
LW-LIC	− 1.123 (0.169)	− 2.103 (0.209)	− 1.613 (0.184)	− 1.453 (0.183)
HW-HIC	− 1.804 (0.169)	− 2.606 (0.209)	− 0.955 (0.184)	− 1.082 (0.183)
HW-LIC	− 1.695 (0.169)	− 2.737 (0.209)	− 0.976 (0.184)	− 0.913 (0.183)

**Table 6:** Results of the models for brain response components in experiment 1.

	N170 in left frontal-central scalp				N400 in left frontal-central scalp			
	*b*	*SE*	*t*	*p*	*b*	*SE*	*t*	*p*
Intercept	− 1.542	0.141	− 10.948	< 0.001	− 2.383	0.178	− 13.391	< 0.001
ICF	0.265	0.108	2.451	0.015	− 0.074	0.126	− 0.588	0.557
WF	0.416	0.108	3.848	< 0.001	0.578	0.126	4.572	< 0.001
ICF **×** WF	0.310	0.216	1.435	0.153	0.113	0.253	0.447	0.655

	N170 in parietal-occipital scalp				N400 in parietal-occipital scalp			
	*b*	*SE*	*t*	*p*	*b*	*SE*	*t*	*p*
Intercept	− 1.159	0.132	− 8.769	< 0.001	− 1.179	0.137	− 8.587	< 0.001
ICF	− 0.272	0.148	− 1.833	0.069	− 0.008	0.140	− 0.060	0.953
WF	− 0.387	0.148	− 2.613	0.010	− 0.362	0.140	− 2.584	0.011
ICF × WF	− 0.500	0.296	− 1.688	0.094	− 0.355	0.280	− 1.266	0.208

Bayes factor analyses for FRP measures were conducted to support the null interactions on N170 and N400. The *BF* values (*BF* = *BF*_Full_/*BF*_main_) were evaluated to test the null interaction hypothesis. The full models (*BF*_*Full*_) contained the main word frequency effect, initial constituent character frequency, and their interactions. In contrast, the main models (*BF*_Main_) contained only the main effects of word frequency and initial constituent character frequency. The BF values over the left frontal-central and parietal-occipital regions were 0.027 and 0.298, respectively, for N170, while they were 0.001 and 0.832, respectively, for N400. Thus, our BF values result generally supported the null hypothesis.

Each effect of word frequency, end character frequency, and their interaction was tested across eight measures (including 4 eye tracking measures and 4 brain response measures). Bonferroni corrections should be used to validate our results^47^. Therefore, an alpha of 0.006 was appropriate in this context (new a = 0.05/8). As seen from Tables 4 and 6, the main effects of word frequency and initial constituent character frequency met the Bonferroni correction standard; however, their interactions did not. The results of the *BF* values from Bayes factor analyses generally supported the no reliable interaction.

## Experiment 2

### Design and stimuli

The experiment utilized a 2 (word frequency: low vs. high) × 2 (end constituent character frequency: low versus high) within-subject design. The sentence materials were prepared using steps similar to those in Experiment 1. A total of 160 sentence frames were constructed to embed the low-frequency low-end character (LW-LEC), low-frequency high-end character (LW-HEC), high-frequency low-end character (HW-LEC), and high frequency high-end character (HW-HEC) target words (Table 7). The mean frequency values, contextual predictability of target words, and sentence naturalness were matched or balanced using standards similar to those used in Experiment 1. The strokes of initial and end characters, along with the frequency of initial characters of target words, were balanced across the four experiment conditions (*ps* > 0.05). The pairs of LW-LEC and LW-HEC, as well as HW-LEC and HW-HEC, were matched on word frequency (*ps* > 0.05), and the pairs of LW-LEC and HW-LEC, as well as LW-HEC & HW-HEC, were matched on initial character frequency (*ps* > 0.05).

**Table 7:** Example materials used in experiment 2.

Conditions	Translations of example materials
LW-LEC	Professor Li will talk about the basic concept of *reform* in this class.
LW-HEC	Professor Li will talk about the basic concept of *cognition* in this class
HW-LEC	Professor Li will talk about the basic concept of *consciousness* in this class
HW-HEC	Professor Li will talk about the basic concept of *motivation* in this class

*LW* low word frequency, *HW* high word frequency, *LEC* low end constituent character frequency, *HEC* high end constituent character frequency.

Target words are in italics. Each sentence can accommodate either a LW-LEC, LW-HEC, HW-LEC or HW-HEC target word.

Twenty college students, native speakers of Chinese who did not participate in either of the two experiments, were asked to rate contextual predictability using a cloze task. There were no differences in the cloze scores among the four conditions *F* (1, 636) *=* 0.112, *p =* 0.953. An additional 40 college students, also native speakers of Chinese, were asked to rate the naturalness of the sentences on a 5-point Likert scale (1 = completely unnatural; 5 = completely natural). No reliable differences were observed among the four conditions *F* (1, 636) *=* 1.242, *p =* 0.293. The mean values mentioned above are presented in Table 8.

**Table 8:** Specifications of target words and the naturalness of sentences used in experiment 2.

Conditions	Word frequency	First character frequency	Second character frequency	First character stroke	Second character stroke	Cloze	Sentences naturalness
LW-LEC	3 (2)	1123 (2351)	172 (318)	7.43 (2.52)	7.59 (2.16)	0.06 (0.56)	4.89 (0.10)
LW-HEC	3 (1)	1028 (2083)	2648 (1962)	7.66 (2.76)	7.48 (2.57)	0.06 (0.79)	4.87 (0.14)
HW-LEC	94 (76)	1069 (1228)	2448 (116)	7.47 (2.68)	7.61 (2.40)	0.09 (0.68)	4.86 (0.12)
HW-HEC	102 (122)	1137 (1602)	2676(3354)	7.61(2.85)	7.53(2.33)	0.09(0.68)	4.87(0.11)

Mean values are shown with standard deviations in parentheses.

*LW* low word frequency, *HW* high word frequency, *LEC* low end constituent character frequency, *HEC* high end constituent character frequency.

### Results

#### Eye tracking results

The means, *SEs*, and statistical effects are presented in Tables 9 and 10. We observed reliable word frequency effects on eye-tracking behaviors, with low-frequency words being fixated on longer, skipped less often, and refixated more frequently than high-frequency words, consistent with the findings of Experiment 1. Moreover, reliable inhibiting effects of end constituent character frequency were reported, with words that have high-frequency end constituent characters being fixated on longer times than those with low-frequency end constituent characters. This finding contradicted a previous study that reported facilitating end character frequency impacts on eye tracking measures^25^. Non-reliable interactions of word frequency and end character frequency were observed in any eye-tracking measure.

**Table 9:** The mean and standard errors of eye movement dependent in experiment 2.

Measure	Low frequency word		High frequency word	
	LEC	HEC	LEC	HEC
First fixation duration	243 (2)	251 (2)	242 (2)	247 (2)
Gaze duration	286 (3)	300 (3)	272 (3)	277 (3)
Word skipping probability	17.8 (0.8)	17.8 (0.8)	21.0 (0.8)	19.8 (0.8)
Word refixation probability	16.5 (0.7)	17.5 (0.7)	11.7 (0.7)	12.1 (0.7)

**Table 10:** Statistical results of eye movement dependents in experiment 2.

	First fixation duration				Gaze duration			
	*b*	*SE*	*t*	*p*	*b*	*SE*	t	*p*
Intercept	245.876	4.905	50.128	< 0.001	286.801	7.249	39.567	< 0.001
ECF	− 7.219	2.487	− 2.902	0.004	− 11.097	3.665	− 3.028	0.002
WF	3.031	2.090	1.450	0.147	19.643	3.060	6.419	< 0.001
ECF × WF	− 2.639	4.173	− 0.632	0.5271	− 8.411	6.110	− 1.377	0.169

	Word skipping probability				Word refixation probability			
	*b*	*SE*	*Z*	*p*	*b*	*SE*	*Z*	*p*
Intercept	− 1.733	0.135	− 12.838	< 0.001	− 1.997	0.102	− 19.538	< 0.001
ECF	0.045	0.057	0.784	0.433	− 0.057	0.062	− 0.911	0.363
WF	− 0.195	0.057	− 3.399	< 0.001	0.446	0.063	7.123	< 0.001
ECF × WF	− 0.082	0.114	− 0.715	0.475	− 0.026	0.125	− 0.212	0.832

#### FRP results

**Fig. 3:**
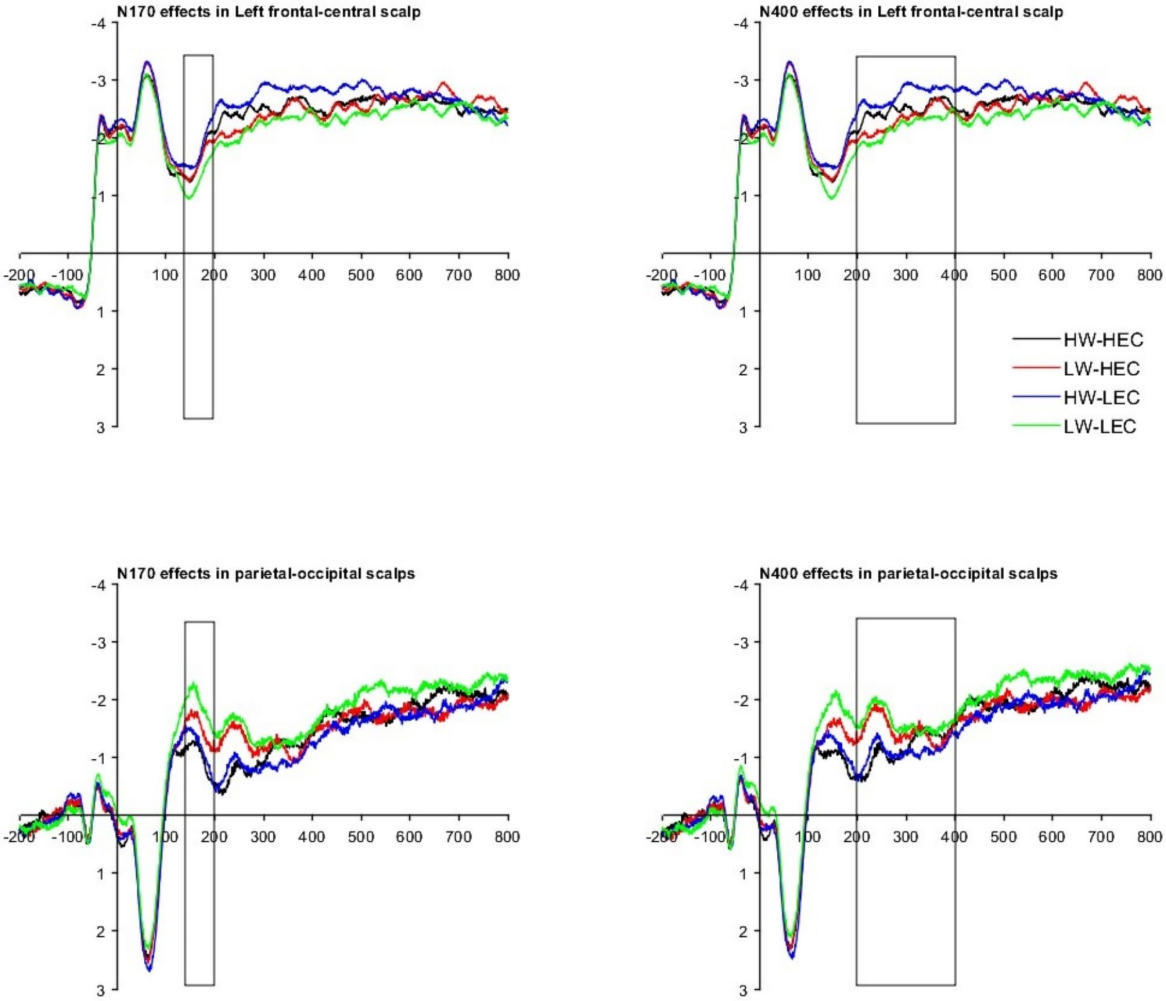
Mean fixation-related potential waveform for four conditions in two interest regions for Experiment 2.

Figure 3; Tables 11 and 12 show the FRP statistical reports for Experiment 2. Word frequency effects on N170 and N400 amplitudes were similar to those found in Experiment 1. Reliable interactions were observed on N170 and N400 over the left frontal-central region. Increased end constituent character frequency effects were found on both N170 and N400 for low-frequency words, in contrast to decreased end constituent character frequency effects for high-frequency words.

**Table 11:** Mean amplitude standard errors of brain response components in experiment 2.

Measure	Left frontal-central scalp		Parietal-occipital scalp	
	N170	N400	N170	N400
LW-HEC	− 1.577 (0.159)	− 2.339 (0.203)	− 1.361 (0.217)	− 1.367 (0.198)
LW-LEC	− 1.267 (0.159)	− 2.149 (0.203)	− 1.709 (0.217)	− 1.558 (0.198)
HW-HEC	− 1.615 (0.159)	− 2.413 (0.203)	− 0.863 (0.217)	− 1.113 (0.198)
HW-LEC	− 1.778 (0.159)	− 3.073 (0.203)	− 1.018 (0.217)	− 1.027 (0.198)

**Table 12:** Results of the models for brain response components in experiment 2.

	N170 in left frontal-central scalp				N400 in left frontal-central scalp			
	*b*	*SE*	*t*	*p*	*b*	*SE*	*t*	*p*
Intercept	− 1.559	0.122	− 12.767	< 0.001	− 2.494	0.162	− 15.388	< 0.001
ECF	0.0736	0.118	0.624	0.533	− 0.236	0.1408	− 1.673	0.096
WF	0.274	0.118	2.328	0.021	0.499	0.1408	3.544	< 0.001
ECF × WF	0.473	0.236	2.006	0.047	0.851	0.2816	3.020	0.003

	N170 in parietal-occipital scalp				N400 in parietal-occipital scalp			
	*b*	*SE*	*t*	*p*	*b*	*SE*	*t*	*p*
Intercept	− 1.238	0.149	− 8.300	< 0.001	− 1.266	0.143	− 8.837	< 0.001
ECF	− 0.252	0.182	− 1.383	0.169	− 0.053	0.158	− 0.332	0.740
WF	− 0.594	0.182	− 3.263	0.001	− 0.393	0.158	− 2.480	0.014
ECF × WF	− 0.193	0.364	− 0.530	0.597	− 0.278	0.3167	− 0.879	0.381

According to Bonferroni corrections^47^, the main effect of word frequency, end constituent character frequency, and their interaction met the standard for statistical significance. The significant interactions of word frequency with end constituent character frequency suggest that word frequency moderates the end character effects.

## General discussion

Based on the synchronous recording of EEG and eye behaviors, the co-registration technique offers more detailed insights into word processing than the eye tracking method alone^38,48^. This approach allows readers to read at their own pace, enhancing the ecological validity of brain responses and reading processes. Utilizing this technique, we explored the processing of words and their constituent characters in Chinese natural silent reading.

### Word frequency effects

Based on previous studies, we replicated the word frequency effects on eye tracking data, with word frequency decreasing first fixation duration, gaze duration, and re-fixation probability and increasing skipping probability^24–27^. By including EEG data, our study enriched the evidence that Chinese visual word access occurs early, which has been consistently demonstrated in ERP studies^9,10,28^. Consistent and reliable word frequency effects were observed for N170 over the parietal-occipital region in both experiments, with lower frequency words eliciting larger negativity. These findings suggest that word access in reading occurred before the stimuli were fixated on for less than 200 ms. Regarding N400 effects, word frequency effects vanished when word presentation rates were similar to reading^37^. Kretzschmar et al. found no N400 word frequency effects during free view reading^44^. However, unlike previous research on alphabetic script reading, we consistently observed reliable N400 effects over the parietal-occipital region in our two experiments.

In both Experiments 1 and 2, inhibitory word frequency effects on brain responses over frontal-central region were consistently observed, with higher word frequency increasing the size of N170 and N400 as compared to low frequency words. Using fixation-related functional magnetic resonance imaging (fMRI), Desai et al. reported that high-frequency words elicited greater activation than low-frequency words^49^. The frontal region is thought to be associated with the resolution of conflict among multiple competing representations and lexical competition^50,51^. Our finding that high frequency words increase negativity over the frontal-central region suggests that such words automatically activate richer features, associated semantics, and/or more similar concepts than low-frequency words. These inhibitory effects on N400 can also be interpreted that high frequency words are accessed semantically and integrated into context more effectively, thereby enhancing semantic processing during fixation.

### Character frequency effects

Robust and consistent inhibiting effects were observed for both initial or end character frequency, with high initial or end constituent character frequency words receiving longer fixation than low initial or end character frequency words. This contrasts with previous eye tracking studies that indicated that character frequency facilitated the processing of Chinese two-character words^25,26^. Inhibiting effects from initial character frequency aligns with findings from recent eye tracking studies^29,30,34^. Our findings regarding end character frequency effects contribute additional evidence of inhibitory character frequency impacts on word decoding. In general, words with higher-frequency characters had more high-frequency neighbors, thus resulting in greater inhibitory effects^31,50^. Because orthographic neighborhood size factors were used as covariates in our models to ensure that they did not affect the results, it can be confirmed that constituent character frequency factors have inhibitory impacts on decoding Chinese multi-character words.

Our findings on N170 and N400 character frequency effects are noteworthy. Our results contribute to reconciling the inconsistent findings of previous eye tracking studies^25,26,29,30,34^. We observed that initial character frequency had inhibiting effects on frontal-central N170, and word frequency reduced the inhibiting effects of end character frequency on frontal-central N400. These results of N170 and N400 over the frontal-central scalp suggest that more orthographic or semantic codes were activated and competed with target identification when readers processed words with high-frequency constituent characters rather than words with low-frequency constituent characters. Facilitating effects of initial character frequency on parietal-occipital N170 (though not significant after Bonferroni corrections) suggest that high frequency initial characters could promote the form access of whole words and/or characters to some extent.

### Cross-language differences

Many studies confirmed that word frequency decreased N400 in lexical decision tasks^8,9,11,12^. However, these word frequency effects diminish when word presentation rates are similar to reading^13,37,60,61^, and even disappear in free-viewing reading^44^. Our regular, persistent word frequency effects over parietal-occipital scalp areas indicate that these word frequency effects could cause resistance to context processing in Chinese reading. In contrast, the reversed word frequency effect over the frontal-central region suggests that frequent words are more easily accessed and more likely to be integrated into contexts at fixation points compared to infrequent words. Overall, both facilitating and inhibiting word frequency effects were observed in brain response measures, implying that two cognitive mechanisms may support the processing of Chinese two-character words during reading. Our finding of word frequency effects on EEG data indicated a potential language-specific mechanism for Chinese word decoding.

Inhibiting effects of syllable frequency on behavioral and brain responses have been documented in French, Spanish, and German^15–19^, while, facilitating syllable frequency effects have been reported in English^22^. Chinese syllables are mapped into characters with their correspondence being lower than in other languages. The character frequency effects observed in our study were complex. We observed both inhibiting and facilitating effects of character frequency on brain responses. The facilitating effects over the parietal-occipital region may suggest that high frequency characters promote form access. Inhibiting effects of initial character frequency observed over the frontal-central scalp may reflect competitive interactions within the same level of sub-lexical processing. Additionally, the reduction of end character frequency effects on N170 and N400 by word frequency over the frontal-central scalp may indicate that lexical-level processing moderates sub-lexical level processing. These findings suggest the presence of language-dependent mechanisms for syllable and character processing.

### Study limitations

Our study had certain methodological limitations that should be acknowledged. Firstly, the time course of word processing did not closely align with that of the FRP waveform, as processing of any given word can begin from parafoveal vision. Consequently. the FRP waveform may have been influenced by ongoing neural processing from previous events. Secondly, character frequency effects are confounded with orthographic neighborhood size, as words with high-frequency characters commonly have large neighborhoods^33^. Orthographic neighborhood size was used as a covariant variable in the linear mixed-effects models to control for its potential influence on eye tracking results. However, isolating these confounds in FRP measures is challenging because FRP data must be averaged across all trials. Additionally, we cannot provide evidence to refute the possibility that Chinese readers may use character-combining mechanisms for decoding extremely low frequency words, as these words were not included in our experiments. Thirdly, the collapsed localizer we used to select electrodes for analyzing N170 and N400, while suitable in the absence of specific prior research parameters, may not be ideal for all contexts^45^. Using EEG equipment with high spatial density electrode distribution could yield more stable effects in further studies.

## Data Availability

Sequence data that support the findings of this study are available at https://osf.io/46sjh/, or from the corresponding author upon reasonable request. The study design, hypotheses, and analytic plan were not preregistered.

## References

[1] Álvarez, C. J., Carreiras, M. & Taf, M. Syllables and morphemes: contrasting frequency effects in Spanish. *J. Exp. Psychol. Learn. Mem. Cogn.* **27** (2), 545–555. https://doi.org/10.1037/0278-7393.27.2.545 (2001). 11294448 10.1037/0278-7393.27.2.545

[2] Carreiras, M., Mechelli, A. & Price, C. J. Effect of word and syllable frequency on activation during lexical decision and reading aloud. *Hum. Brain Mapp.* **27** (12), 963–972. https://doi.org/10.1002/hbm.20236 (2006). 16628608 10.1002/hbm.20236 PMC3261381

[3] Conrad, M. & Jacobs, A. M. Replicating syllable frequency effects in Spanish in German: one more challenge to computational models of visual word recognition. *Lang. Cogn. Process.* **19** (3), 369–390. https://doi.org/10.1080/01690960344000224 (2004).

[4] Hand, C. J., Miellet, S., ODonnell, P. J. & Sereno, S. C. The frequency-predictability interaction in reading: it depends where you’re coming from. *J. Exp. Psychol. Hum. Percept. Perform.* **36** (5), 1294–1313. https://doi.org/10.1037/a0020363 (2010). 20854004 10.1037/a0020363

[5] Brysbaert, M., Mandera, P. & Keuleers, E. The word frequency effect in word processing: an updated review. *Curr. Dir. Psychol. Sci.* **21** (1), 45–50. 10.1177/0963721417727521 (2018).

[6] Rayner, K., Ashby, J., Pollatsek, A. & Reichle, E. D. The effects of frequency and predictability on eye fixations in reading: implications for the E-Z reader model. *J. Exp. Psychol. Hum. Percept. Perform.* **30**, 720–732. https://doi.org/10.1037/0096-1523.30.4.720 (2004). 15301620 10.1037/0096-1523.30.4.720

[7] White, S. J., Drieghe, D., Liversedge, S. P. & Staub, A. The word frequency effect during sentence reading: a linear or nonlinear effect of log frequency? *Q. J. Exp. Psychol.* **71** (1), 46–55. https://doi.org/10.1080/17470218.2016.1240813 (2016). 10.1080/17470218.2016.1240813 27760490

[8] Barber, H., Vergara, M. & Carreiras, M. Syllable-frequency effects in visual word recognition: evidence from ERPs. *Neuroreport* **15** (3), 545–548. https://doi.org/10.1097/00001756-200403010-00032 (2004) 15094520 10.1097/00001756-200403010-00032

[9] Hauk, O. & Pulvermüller, F. Effects of word length and frequency on the human event-related potential. *Clin. Neurophysiol.* **115**, 1090–1103. https://doi.org/10.1016/j.clinph.2003.12.020 (2004). 15066535 10.1016/j.clinph.2003.12.020

[10] Dambacher, M., Kliegl, R., Hofmann, M. & Jacobs, A. M. Frequency and predictability effects on event-related potentials during reading. *Brain Res.* **1084** (1), 89–103. 10.1016/j.brainres.2006.02.010 (2006). 16545344 10.1016/j.brainres.2006.02.010

[11] Conrad, M., Tamm, S., Carreiras, M. & Jacobs, A. Simulating syllable frequency effects within an interactive activation framework. *Eur. J. Cogn. Psychol.* **22**, 861–893. https://doi.org/10.1080/09541440903356777 (2010).

[12] Grainger, J., Lopez, D., Eddy, M., Dufau, S. & Holcomb, P. J. How word frequency modulates masked repetition priming: an ERP investigation. *Psychophysiology* **49**, 604–616. https://doi.org/10.1111/j.1469-8986.2011.01337.x (2012). 22221077 10.1111/j.1469-8986.2011.01337.x PMC3323680

[13] Kutas, M. & Hillyard, S. A. Reading senseless sentences: brain potentials reflect semantic incongruity. *Science* **207**, 203–205. https://doi.org/10.1126/science.7350657 (1980). 7350657 10.1126/science.7350657

[14] Federmeier, K. D. Connecting and considering: Electrophysiology provides insights into comprehension. *Psychophysiology* **59** (1), e13940. 10.1111/psyp.13940 (2022). 34520568 10.1111/psyp.13940 PMC9009268

[15] Conrad, M., Grainger, J. & Jacobs, A. Phonology as the source of syllable frequency effects in visual word recognition: evidence from French. *Mem. Cognit*. **35**, 974–983. https://doi.org/10.3758/bf03193470 (2007). 17910181 10.3758/bf03193470

[16] Conrad, M., Carreiras, M., Tamm, S. & Jacobs, A. Syllables and bigrams: orthographic redundancy and syllabic units affect visual word recognition at different processing levels. *J. Exp. Psychol. Hum. Percept. Perform.* **35** (2), 461–479. https://doi.org/10.1037/a0013480 (2009). 19331501 10.1037/a0013480

[17] Hawelka, S., Schuster, S., Gagl, B. & Hutzler, F. Beyond single syllables: the effect of first syllable frequency and orthographic similarity on eye movements during silent reading. *Lang. Cogn. Process.* **28** (8), 1134–1153. https://doi.org/10.1080/01690965.2012.696665 (2013).

[18] Mahé, G., Bonnefond, A. & Doignon-Camus, N. The time course of the syllable frequency effect in visual word recognition: evidence for both facilitatory and inhibitory effects in French. *Read. Writ.* **27**, 171–187. https://doi.org/10.1007/s11145-013-9438-3 (2014).

[19] Montani, V., Chanoine, V., Grainger, J. & Ziegler, J. C. Frequency-tagged visual evoked responses track syllable effects in visual word recognition. *Cortex* **121**, 60–77. https://doi.org/10.1016/j.cortex.2019.08.014 (2019). 31550616 10.1016/j.cortex.2019.08.014

[20] Hutzler, F. et al. Inhibitory effects of first syllable-frequency in lexical decision: an event related potential study. *Neurosci. Lett.* **372**, 179–184. https://doi.org/10.1016/j.neulet.2004.07.050 (2004). 15542236 10.1016/j.neulet.2004.07.050

[21] Hutzler, F., Conrad, M. & Jacobs, A. M. Effects of syllable-frequency in lexical decision and naming: An eye movement study. *Brain Lang.* **92** (2), 138–152. https://doi.org/10.1016/j.bandl.2004.06.001 (2005). 15629488 10.1016/j.bandl.2004.06.001

[22] Macizo, P. & Van Petten, C. Syllable frequency in lexical decision and naming of English words. *Read. Writ.* **20** (4), 295–331. https://doi.org/10.1007/s11145-006-9032-z (2007).

[23] Croot, K. et al. Syllable frequency effects in immediate but not delayed syllable naming in English. *Lang. Cogn. Neurosci.* **32** (9), 1119–1132. https://doi.org/10.1080/23273798.2017.1284340 (2017).

[24] Li, X., Bicknell, K., Liu, P., Wei, W. & Rayner, K. Reading is fundamentally similar across disparate writing systems: a systematic characterization of how words and characters influence eye movements in Chinese reading. *J. Exp. Psychol. Gen.* **143** (2), 895–913. https://doi.org/10.1037/a0033580 (2014). 23834023 10.1037/a0033580 PMC3885613

[25] Yan, G., Tian, H., Bai, X. & Rayner, K. The effect of word and character frequency on the eye movements of Chinese readers. *Br. J. Psychol.* **97**, 259–268. https://doi.org/10.1348/000712605X70066 (2006). 16613652 10.1348/000712605X70066

[26] Ma, G., Li, X. & Rayner, K. Readers extract character frequency information from non-fixated target word at long pretarget fixations during Chinese reading. *J. Exp. Psychol. Hum. Percept. Perform.* **41** (5), 1409–1419. 10.1037/xhp0000072 (2015). 26168144 10.1037/xhp0000072 PMC4767270

[27] Wang, J. et al. Adult age differences in eye movements during reading: the evidence from Chinese. *J. Gerontol. B Psychol. Sci. Soc. Sci.* **73**, 584–593. https://doi.org/10.1093/geronb/gbw036 (2018). 27032427 10.1093/geronb/gbw036 PMC6019021

[28] Lee, C. Y., Liu, Y. N. & Tsai, J. L. The time course of contextual effects on visual word recognition. *Front. Psychol.* **3**, 285. 10.3389/fpsyg.2012.00285 (2012). 22934087 10.3389/fpsyg.2012.00285 PMC3422729

[29] Yu, L., Liu, Y., Reichle, E. D. A. & Corpus-Based Versus Experimental examination of Word- and character-frequency effects in Chinese Reading: theoretical implications for models of Reading. *J. Exp. Psychol. Gen.* **150** (8), 1612–1641. https://doi.org/10.1037/xge0001014 (2021). 33332143 10.1037/xge0001014

[30] Xiong, J., Yu, L., Veldre, A., Reichle, E. D. & Andrews, S. A multitask comparison of word- and character-frequency effects in Chinese reading. *J. Exp. Psychol. Learn. Mem. Cogn.* **49** (5), 793–811. https://doi.org/10.1037/xlm0001192 (2023). 36326651 10.1037/xlm0001192

[31] Huang, H. W. et al. Orthographic neighborhood effects in reading Chinese two-character words. *Neuroreport* **17** (10), 1061–1065. https://doi.org/10.1097/01.wnr.0000224761.77206.1d (2006). 16791104 10.1097/01.wnr.0000224761.77206.1d

[32] Tsang, Y. K. et al. MELD-SCH: a megastudy of lexical decision in simplified Chinese. *Behav. Res. Methods*. **50**, 1763–1777. 10.3758/s13428-017-0944-0 (2018). 28779457 10.3758/s13428-017-0944-0

[33] Tsang, Y. K. & Zou, Y. An ERP megastudy of Chinese word recognition. *Psychophysiology* **59** (11), e14111. https://doi.org/10.1111/psyp.14111 (2022). 35609148 10.1111/psyp.14111

[34] Cui, L. et al. Compound word frequency modifies the effect of character frequency in reading Chinese. *Q. J. Exp. Psychol.* **74** (4), 610–633. https://doi.org/10.1177/1747021820973661 (2021). 10.1177/1747021820973661 PMC8044629 33118461

[35] Penolazzi, B., Hauk, O. & Pulvermüller, F. Early semantic context integration and lexical access as revealed by event-related brain potentials. *Biol. Psychol.* **74**, 374–388. https://doi.org/10.1016/j.biopsycho.2006.09.008 (2007). 17150298 10.1016/j.biopsycho.2006.09.008

[36] Dambacher, M. et al. Stimulus onset asynchrony and the timeline of word recognition: event-related potentials during sentence reading. *Neuropsychologia* **50**, 1852–1870. https://doi.org/10.1016/j.neuropsychologia.2012.04.011 (2012). 22564485 10.1016/j.neuropsychologia.2012.04.011

[37] Hutzler, F. et al. Welcome to the real world: validating fixation-related brain potentials for ecologically valid settings. *Brain Res.* **1172**, 124–129. https://doi.org/10.1016/j.brainres.2007.07.025 (2007). 17803976 10.1016/j.brainres.2007.07.025

[38] Metzner, P., von der Malsburg, T., Vasishth, S. & Rösler, F. Brain responses to World Knowledge violations: a comparison of stimulus- and fixation-triggered event-related potentials and neural oscillations. *J. Cogn. Neurosci.* **27** (5), 1017–1028. https://doi.org/10.1162/jocn_a_00731 (2015). 25269112 10.1162/jocn_a_00731

[39] Degno, F. & Liversedge, S. P. Eye movements and fixation-related potentials in reading: a review. *Vision* **4** (1). 10.3390/vision4010011 (2020). 10.3390/vision4010011 PMC7157570 32028566

[40] Shen, W. & Li, X. S. The uniqueness of word superiority effect in Chinese reading. *Chin. Sci. Bull.* **57** (35), 3414–3420. https://doi.org/10.1360/972012-666 (2012).

[41] Barr, D. J., Levy, R., Scheepers, C. & Tily, H. J. Random effects structure for confirmatory hypothesis testing: keep it maximal. *J. Mem. Lang.* **68**, 255–278. https://doi.org/10.1016/j.jml.2012.11.001 (2013). 10.1016/j.jml.2012.11.001 PMC3881361 24403724

[42] Baayen, R. H., Davidson, D. J. & Bates, D. M. Mixed-effects modeling with crossed random effects for subjects and items. *J. Mem. Lang.* **59**, 390–412. 10.1016/j.jml.2007.12.005 (2008).

[43] Dimigen, O. Optimizing the ICA-based removal of ocular EEG artifacts from free viewing experiments. *NeuroImage* **207**, 116–117. 10.1016/j.neuroimage.2019.116117 (2020). 10.1016/j.neuroimage.2019.116117 31689537

[44] Kretzschmar, F., Schlesewsky, M. & Staub, A. Dissociating word frequency and predictability effects in reading: evidence from coregistration of eye movements and EEG. *J. Exp. Psychol. Learn. Mem. Cogn.* **41** (6), 1648–1662. https://doi.org/10.1037/xlm0000128 (2015). 26010829 10.1037/xlm0000128

[45] Luck, S. J. & Gaspelin, N. How to get statistically significant effects in any ERP experiment (and why you shouldn’t). *Psychophysiology* **54** (1), 146–157. https://doi.org/10.1111/psyp.12639 (2017). 28000253 10.1111/psyp.12639 PMC5178877

[46] Cai, Q. & Brysbaert, M. S. U. B. T. L. E. X. C. H. Chinese word and character frequencies based on film subtitles. *PLoS One*. **5** (6), e10729. 10.1371/journal.pone.0010729 (2010). 20532192 10.1371/journal.pone.0010729 PMC2880003

[47] von der Malsburg, T. & Angele, B. False positives and other statistical errors in standard analyses of Eye Movements in Reading. *J. Mem. Lang.* **94**, 119–133. https://doi.org/10.1016/j.jml.2016.10.003 (2017). 28603341 10.1016/j.jml.2016.10.003 PMC5461930

[48] Dimigen, O., Sommer, W., Hohlfeld, A., Jacobs, A. M. & Kliegl, R. Coregistration of eye movements and EEG in natural reading: analyses and review. *J. Exp. Psychol. Gen.* **140** (4), 552–572. https://doi.org/10.1037/a0023885 (2011). 21744985 10.1037/a0023885

[49] Desai, R. H., Choi, W. & Henderson, J. M. Word frequency effects in naturalistic reading. *Lang. Cogn. Neurosci.* **35** (5), 583–594. 10.1080/23273798.2018.1527376 (2020). 33015218 10.1080/23273798.2018.1527376 PMC7531031

[50] Schnur, T. T. et al. Localizing interference during naming: convergent neuroimaging and neuropsychological evidence for the function of Broca’s area. *Proc. Natl. Acad. Sci. U S A*. **106** (1), 322–327. 10.1073/pnas.080587410 (2009). 19118194 10.1073/pnas.0805874106 PMC2629229

[51] Han, S., O’Connor, A. R., Eslick, A. N. & Dobbins, I. G. The role of left ventrolateral prefrontal cortex during episodic decisions: semantic elaboration or resolution of episodic interference? *J. Cogn. Neurosci.* **24** (1), 223–234. https://doi.org/10.1162/jocn_a_00133 (2012). 21916561 10.1162/jocn_a_00133 PMC3417066

[52] Yao, P., Staub, A. & Li, X. Predictability eliminates neighborhood effects during Chinese sentence reading. *Psychon Bull. Rev.* **29** (1), 243–252. 10.3758/s13423-021-01966-1 (2021). 34258731 10.3758/s13423-021-01966-1

[53] Carreiras, M., Álvarez, C. & Devega, M. Syllable frequency and visual word recognition in Spanish. *J. Mem. Lang.* **32** (6), 766–780. https://doi.org/10.1006/jmla.1993.1038 (1993).

[54] Rao, R. & Ballard, D. Predictive coding in the visual cortex: a functional interpretation of some extra-classical receptive-field effects. *Nat. Neurosci.* **2**, 79–87. https://doi.org/10.1038/4580 (1999). 10195184 10.1038/4580

[55] Friston, K. The free-energy principle: a unified brain theory? *Nat. Rev. Neurosci.* **11**, 127–138. https://doi.org/10.1038/nrn2787 (2010). 20068583 10.1038/nrn2787

[56] Price, C. J. & Devlin, J. T. The interactive account of ventral occipitotemporal contributions to reading. *Trends Cogn. Sci.* **15** (6), 246–253. 10.1016/j.tics.2011.04.001 (2011). 21549634 10.1016/j.tics.2011.04.001 PMC3223525

[57] Lewis, A. G. & Bastiaansen, M. A predictive coding framework for rapid neural dynamics during sentence-level language comprehension. *Cortex* **68**, 155–168. https://doi.org/10.1016/j.cortex.2015.02.014 (2015). 25840879 10.1016/j.cortex.2015.02.014

[58] Kuperberg, G. R. & Jaeger, T. F. What do we mean by prediction in language comprehension? *Lang. Cogn. Neurosci.* **31** (1), 32–59. https://doi.org/10.1080/23273798.2015.1102299 (2016). 27135040 10.1080/23273798.2015.1102299 PMC4850025

[59] Jiang, L. P. & Rao, R. Predictive coding theories of cortical function. *arXive-prints*10.48550/arXiv.2112.10048 (2021).

[60] Tune, S. et al. Cross-linguistic variation in the neurophysiological response to semantic processing: evidence from anomalies at the borderline of awareness. *Neuropsychologia* **56**, 147–166. https://doi.org/10.1016/j.neuropsychologia.2014.01.007 (2014). 24447768 10.1016/j.neuropsychologia.2014.01.007 PMC3966966

[61] Sereno, S. C., Hand, C. J., Shahid, A., Mackenzie, I. G. A. & Leuthold, H. Early EEG correlates of word frequency and contextual predictability in reading. *Lang. Cogn. Neurosci.* **35** (5), 625–640. 10.1080/23273798.2019.1580753 (2020).

